# Supplementary figures and images for: Protein Translocation Control in E. coli via Temperature-Dependent Aggregation: Application to a Conditionally Lethal Enzyme, Levansucrase
Source: Biomolecules. 2025 Aug 20;15(8):1199. doi: 10.3390/biom15081199 (PMC12384325; doi:10.3390/biom15081199)

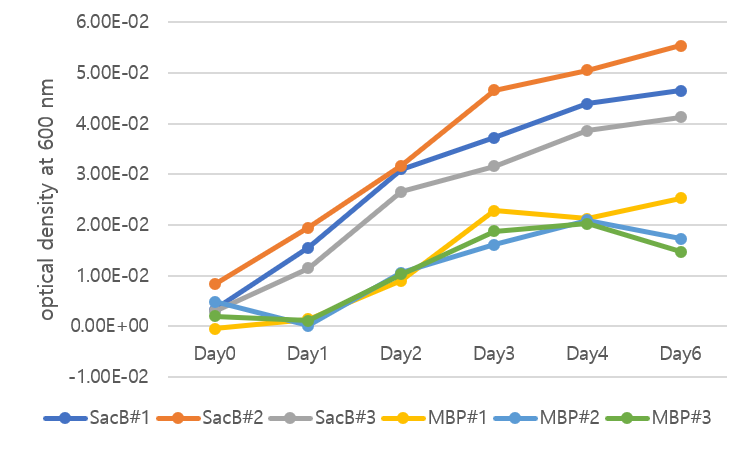

Supplement: Supplementary file 1 [file biomolecules-15-01199-s001.zip › FigureS1-turbidityAssay@600nm.png]
